# Supplementary material for: The influencing factors for distribution patterns of resident and migrant bird species richness along elevational gradients
Source: PeerJ. 2022 Apr 29;10:e13258. doi: 10.7717/peerj.13258 (PMC9059752; doi:10.7717/peerj.13258)
Supplement: Supplemental Information 1 — Significant paths (P < 0.05) are indicated in bold. Critical value indicate the strength of the relationship. [file peerj-10-13258-s001.pdf]

Table S1. The summary of piecewise structural equation modelling (pSEM) for testing of the hypotheses of resident and migrant distribution. Significant paths (*P*<0.05) are indicated in bold. Critical value indicate the strength of the relationship.

Processes	Predictor variable	Response variable	Estimate	Critical value	*P*-value	SE
Common	Elevation	Mean spring temperature	-0.69	-13.94	**<0.001**	-0.004
	Elevation	Understory vegetation	0.34	2.57	**0.011**	0.0009
	Elevation	Overstory vegetation	0.14	1.67	0.096	0.0004
	Elevation	Habitat diversity	-0.68	-5.84	**<0.001**	-0.001
Hypotheses regarding resident species richness	Mean temperature	Resident species richness	0.32	2.27	**0.025**	0.36
	Understory vegetation	Resident species richness	0.07	0.80	0.426	0.16
	Overstory vegetation	Resident species richness	0.16	1.79	0.076	0.38
	Habitat diversity	Resident species richness	0.13	1.12	0.263	0.54
Hypotheses regarding migrant species richness	Mean temperature	Migrant species richness	-0.48	-4.86	**<0.001**	-0.41
	Understory vegetation	Migrant species richness	0.11	1.44	0.159	0.21
	Overstory vegetation	Migrant species richness	0.34	4.85	**<0.001**	0.60
	Habitat diversity	Migrant species richness	0.14	1.46	0.147	0.43
