# Supplementary material for: The influencing factors for distribution patterns of resident and migrant bird species richness along elevational gradients
Source: PeerJ. 2022 Apr 29;10:e13258. doi: 10.7717/peerj.13258 (PMC9059752; doi:10.7717/peerj.13258)
Supplement: Supplemental Information 2 — Bolds indicate a significant relationship between variables. [file peerj-10-13258-s002.pdf]

Table S2. Simple linear regressions according to the hypotheses of processes. Bolds indicate a significant relationship between variables.

Processes	Response variable	predictor variable	Coefficient (β)	R^2	F	*P*-value
Hypotheses regarding resident species richness	Resident species richness	Mean temperature	0.43±0.090	0.138	22.51	**<0.001**
		Understory vegetation	-0.08±0.202	0.001	0.162	0.688
		Overstory vegetation	0.25±0.196	0.011	1.671	0.198
		Habitat diversity	1.23±0.344	0.083	12.75	**<0.001**
Hypotheses regarding migrant species richness	Migrant species richness	Mean temperature	-0.41±0.063	0.237	43.49	**<0.001**
		Understory vegetation	0.43±0.147	0.057	8.508	**0.004**
		Overstory vegetation	0.70±0.134	0.164	27.4	**<0.001**
		Habitat diversity	-0.86±0.258	0.073	11.07	**0.001**
